# Outcomes after bone grafting in patients with and without ACL revision surgery: a retrospective study

**DOI:** 10.1186/s12891-018-2174-8

**Published:** 2018-07-21

**Authors:** Theresa Diermeier, Elmar Herbst, Sepp Braun, Emine Saracuz, Andreas Voss, Andreas B. Imhoff, Andrea Achtnich

**Affiliations:** Department of Orthopaedic Sports Medicine, Klinikum rechts der Isar, TU Munich, Ismaninger Str. 22, 81675 Munich, Germany

**Keywords:** ACL reconstruction, Autologous bone grafting, Revision ACL surgery, Recurrence

## Abstract

**Background:**

Current literature is lacking of data regarding functional outcomes in patients following bone tunnel grafting with or without revision anterior cruciate ligament (ACL) reconstruction. Therefore, the aim of the present study was to evaluate the clinical outcome in patients with (RACL) or without revision ACL reconstruction (OBG) following bone grafting.

**Methods:**

Fifty-nine patients (18 female, 41 male) who underwent bone grafting due to recurrent, symptomatic ACL deficiency following ACL reconstruction between 2011 and 2014 were retrospectively analyzed. In 44 patients (mean age: 30,5 ± 8,5 years) a staged revision ACL reconstruction (RACL) was performed after bone grafting. 10 patients (mean age: 33.2 ± 10.3 years) refused to have ACL revision surgery after bone grafting (OBG). Outcome measures included instrumented laxity testing, the International Knee Documentation Committee (IKDC) score, the Knee Injury and Osteoarthritis Outcome Score (KOOS), Lysholm score and Tegner activity scale.

**Results:**

After mean period of 33,9 ± 17.0 months, 54 patients were available for follow up examination. In the RACL group, the Lysholm score was 77,2 ± 15,5 (range 35–100), the mean IKDC subjective knee score was 69,0 ± 13,4 (range 39,1–97,7) and the mean Tegner activity score was 4,1 ± 1,5 (range, 1–9). Similarly, in the OBG group the mean Lysholm score was 72,90 ± 18,7 (range 50–100), the mean IKDC subjective score was 69,3 ± 20,0 (range 44,1–100) and the mean Tegner activity score was 4,6 ± 1,2 (range, 3–6). No significant difference was observed between the two groups. Knee laxity measurements were elevated without revision ACL surgery, however the difference was not significant.

**Conclusion:**

Bone tunnel grafting with or without second stage ACL revision surgery showed no significant difference in functional outcome score. Thus, in case of revision ACL instability careful patient selection is necessary and expectations should be discussed openly with the patients.

## Background

Revision anterior cruciate ligament (ACL) reconstruction is continuously increasing with a current incidence of up to 15% [1–3]. Younger age, secondary trauma, insufficient graft healing and technical factors such as graft fixation or position were associated with increased failure rate after primary ACL reconstruction [4].

In patients requiring revision ACL reconstruction, either a single-stage or a two-stage procedure is indicated based on tunnel position (especially partially anatomic tunnels) and width as well as other potential risk factors, such as lower limb malalignment and steep posterior tibial slope.

In contrast to a single-stage ACL revision, a two-stage procedure consists of 1) hardware removal and graft remnant debridement and bone tunnel grafting and 2) revision ACL reconstruction.

Although two-stage revision ACL reconstruction could achieve similar laxity measurement compared to primary ACL reconstruction, functional outcome parameters have generally been shown to be inferior [2, 5]. From the patient’s perspective especially a two-stage surgery requires high commitment the expectations should be set properly. Clinically, a subset of patients refuses to have further ACL revision surgery despite an already performed bone grafting.

Current literature is lacking of studies evaluating the clinical outcome in patients with and without revision ACL reconstruction following bone grafting. Therefore, the purpose of this study was to evaluate the clinical outcome in patients with and without revision ACL surgery following bone grafting. It was hypothesized that patient reported outcomes are comparable, while knee laxity measures are inferior in patients without revision ACL surgery.

## Methods

In this retrospective study, patients who underwent bone tunnel grafting with autologous bone from iliac crest due to ACL re-injury between January 2011 and December 2014 were included. The decision to perform a two-stage revision was based on computer tomography (CT) scan analysis. Partially anatomic tunnel position combined with tunnel widening > 12 mm, significant tunnel widening (> 12 mm), suboptimal bone stock or inability to place new tunnel properly were indications for a two-stage procedure. Additional inclusion criteria were available, including preoperative radiographs (a.p. and lateral), CT scans of the involved knee and age > 18 years. Patients with multiple ligament instability requiring surgical treatment or missing data from primary ACL reconstruction were excluded. Demographic data, date and type of primary ACL surgery were extracted from the medical reports.

### Clinical examination

All patients underwent clinical examination after a mean follow-up period of 33,9 ± 17.0 months (range, 8,0 to 68 months). Clinical evaluation of the patient’s knee joint was based on the International Knee Documentation Committee (IKDC) form. A goniometer was used to measure the range-of-motion (ROM). Knee stability testing included the medial collateral ligament (MCL) and lateral collateral ligament (LCL), as well as the ACL and posterior cruciate ligament (PCL). For the ACL, anterior laxity was evaluated using the KT-1000 at 30° of flexion and Pivot shift test, graded according to the IKDC grading system. Patient reported outcomes included the IKDC, Knee injury and Osteoarthrtitis Outcome Score (KOOS), Tegner and Lysholm Score.

### Surgical technique

All surgical revision procedures were performed in one hospital. The patient was placed in a supine position and the surgery was performed with the patient under general anesthesia. First, a routine arthroscopy through a high anterolateral and an additional anteromedial portal was performed. A probe was used to examine the cruciate ligaments, menisci and the cartilage. Meniscus and cartilage lesions were treated as indicated.

### Bone grafting technique

The remnants from the previous graft, scar tissue and previous fixation material (interference screws, staples, etc.) were removed. Furthermore, tunnel position and diameter were evaluated and the final decision of bone grafting the tunnels was made. To properly localize the center of the femoral tunnel a guide wire was inserted in the previous tunnel. Tunnels were reamed, curretaged and rasped until they were fully cleared from sclerotic bone. An incision was then made within the existing scar on the proximal tibia to clear the tibial tunnel as described above. Then, both tunnel diameters were estimated with the help of a specific guide system (Arthrex OATS system, Naples, FL, USA) and bone cylinders from the ipsilateral iliac crest were harvested and extracted. With a donor guide, the bone cylinders were placed arthroscopically and impacted in the cleared tunnels. Protrusion of the cylinders was excluded by direct visualization. If necessary, the procedure was performed for both tunnels, femoral and tibial. Postoperatively, only partial weight bearing was permitted for the first two weeks with immediate unrestricted ROM..

### Revision ACL surgery

After confirming adequate bone healing on follow-up CT (minimum 3 months postoperatively) the second operation was planned. Graft choice depended on the initial graft used. Following routine arthroscopy, the femoral and tibial bone tunnels were then prepared in a standardized fashion. For tunnel placement, a specific drill guide was used (Arthrex, Naples, FL, USA). Graft fixation was depended of the chosen tendon type. Femorally, the graft was fixed with a resorbable interference screw (bone-to-bone fixation) or for tendon-to-bone fixation with an extra cortical button system (Arthrex TightRope, Naples, FL, USA). Tibial graft fixation was performed with a resorbable interference screw (Arthrex TightRope, Naples, FL, USA.).

### Rehabilitation

Rehabilitation in revision ACL reconstruction comprised wearing a knee brace with unrestricted ROM for the first six weeks. Furthermore only partial weight bearing was permitted for the first for two weeks. Physical therapy with muscle strengthening and proprioceptive training was performed at least 3 months after the first operation.

The study was approved by the institutional review board of the Technical University of Munich (No. 353/ 15) and conducted according to the Declaration of Helsinki. All patients gave their written informed consent to participate in this investigation.

### Statistical analysis

For statistical analysis the significance level was set at *p* < 0,05. Data distribution was evaluated using the Shapiro-Wilk test. As data were not normally distributed, the Mann-Whitney-U test was used to compare patient reported outcome scores between patients who underwent revision ACL surgery and those without revision ACL reconstruction following bone grafting. Laxity measurements were evaluated using the Pearson’s chi-squared test.

## Results

Fifty-nine patients with bone tunnel grafting were included. Five patients were excluded from the study, because they declined participating in the study. Of the remaining 54 patients, 9 declined to participate in follow-up examination, but all of them consented to complete the patient reported outcome scores. In 44 patients a two stage revision ACL reconstruction (RACL) was performed after bone grafting, whereas 10 patients did not undergo further operation after bone grafting (OBG). Primary ACL reconstructions were performed by several orthopedic surgeons and also at various hospitals. After ACL graft re-rupture all patients present with subjective instability and epsiodes of “giving way” in our outpatient clinic. ACL graft re-rupture was confirmed by clinical examination and magnetic resonance imaging (MRI).

In the RACL group consisting of 31 females and 13 males, the mean age at the time of bone grafting was 30,5 ± 8.5 years (range 16.7 to 49.4 years). For revision ACL reconstruction the contralateral hamstring tendons were used in 46.7% (21/45), ipsilateral hamstring tendons in 26.7% (12/45), ipsilateral quadriceps tendon in 22.2% (10/45) and ipsilateral BTB graft in 2.2% (1/45).

The group of patients without revision ACL reconstruction (OBG) after bone tunnel grafting included 7 females and 3 males with a mean patient age of 33.2 ± 10.3 years (range 18.2–47.7 years).

### Meniscus status and cartilage injuries during bone grafting procedure

Concomitant meniscus and cartilage injuries were evaluated during arthroscopy, which was conducted at every patient before the bone grafting procedure (Table 1). Additional lesions were treated in 45% of the RACL group and in 60% of the subjects who remained without revision ACL reconstruction.

**Table 1:** Concomitant meniscus and cartilage lesions which were evaluated during arthroscopy. Arthroscopy was always performed before bone grafting procedure

	Bone grafting without revision ACL (OBG)	Revision ACL Group (RACL)
Medical Meniscus lesion	1 (10.0%)	11 (25.0%)
Lateral Meniscus lesion	5 (50.0%)	8 (18.2%)
Medial cartilage defect	7 (70.0%)	27 (61.4%)
Lateral cartilage defect	3 (30.0%)	10 (22.7%)
Patellofemoral cartilage defect	4 (40.0%)	10 (22.7%)

### Clinical examination

Knee laxity measurements were elevated without revision ACL surgery, but the difference was not significant (NS). Laxity measurements for both groups (RACL and OBG) are summarized in Table 2. Postoperatively no complications were reported. None of the included patients had a flexion or extensions deficit.

**Table 2:** Laxity measurements, side -to-side difference in KT 1000 for group without revision ACL reconstruction (OBG) and patients with revision ACL reconstruction (RACL)

	Bone Grafting Without Revision ACL (OBG)	Revision ACL Reconstruction (RACL)
Side-to-Side Difference (SSD)		
0-2 mm	50,0%	79,5%
3–4 mm	33,3%	15,4%
≥ 5 mm	16,7%	5,1%
Pivot -Shift Test		
-	16,7%	74,4%
+	33,3%	25,6%
++	50,0%	–

### Clinical outcome

**Fig. 1:**
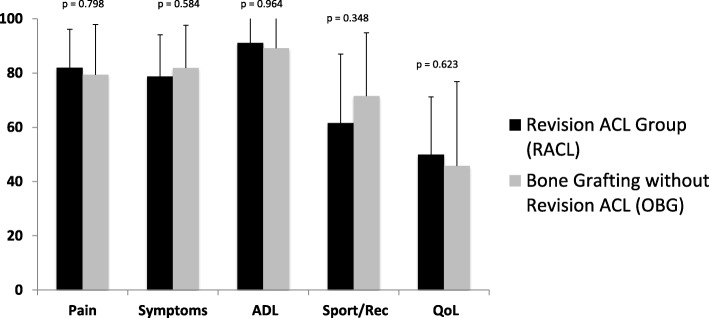
KOOS Score at final follow up for group without revision ACL reconstruction (OBG) and patients with revision ACL reconstruction (RACL). No statistically significant difference was observed between the two groups

At final follow up examination no significant differences were observed between both groups for IKDC (*p* = 0.973), Lysholm (*p* = 0.525) or Tegner activity scale (*p* = 0.326) (Table 3). Likewise, no significant difference was observed for the five KOOS subscales between the groups (Fig. 1).

**Table 3:** Functional outcome at final follow up for group without revision ACL reconstruction (OBG) and patients with revision ACL reconstruction (RACL)

	Bone Grafting Without Revision ACL (OBG) Mean ± SD (Range)	Revision ACL Reconstruction (RACL) Mean ± SD (Range)
IKDC ± SD	69.3 ± 20.0 (Range 44.8–100,0)	69.0 ± 13.4 (Range 39.1–97.7)
Lysholm ± SD	72.9 ± 18.7 (Range 50–100)	77.2 ± 15.5 (Range 35–100)
Tegner ± SD	4.6 ± 1.2 (Range 3–6)	4.1 ± 1.4 (range 1–9)

## Discussion

The main finding of the present study was that after bone tunnel grafting in case of ACL re-instability not every patient requires revision ACL reconstruction. However, there was also no significant difference in the objective clinical outcome between both groups.

Based on our results almost 20% refused a revision ACL reconstruction after bone tunnel grafting, although the initial plan was to perform a revision ACL reconstruction in all patients. Most of these patients have refused to a revision ACL reconstruction due to reduced symptoms after initial bone grafting. At final follow up, laxity measurements were clearly elevated when RACL reconstruction was not performed. However, no significant difference was observed in any of the other evaluated parameters.

The reasons why some patients refused a second ACL reconstruction might be multifactorial. Noyes et al. [6] divided patients with ACL injuries into three different groups depending on their behavior after the injury and the necessity of treatment. “Coper” could achieve recreational level of activity without reconstruction [7]. “Adapter” modified their level of activity until they could manage with the injury. “Non-coper” suffered from recurrent episodes of “giving-way” phenomena and subjective instability during activities of daily living. Therefore in non-coper ACL reconstruction is the treatment of choice [8]. On the other hand, conservative treatment in potential copers also leads to high rate of return to sport and significant reduced instability [9–11]. Interestingly, Moksnes et al. [12] also described non-copers, with the potential to be converted to real copers by non-operative treatment [12]. Although 70% of non-copers were converted to real copers, this proportion might be too high [12]. Nevertheless it demonstrated that non-coper could be changed in to true coper by conservative treatment [11]. In addition, Fitzgerald et al. [9] achieved after standard physical therapy with perturbation training a high return to high-level activity and significant reduced episodes of “giving- way”.

After initial bone grafting, physical therapy with muscle strengthening and proprioceptive training was performed for at least 3 months. However, not only the improved muscle strength is the decisive factor, but the changed functional movement patterns after intensive physical therapy [11]. Therefore, one possible factor, why some patients in the current study rejected revision ACL reconstruction might be due to improved movement pattern. The comparable functional outcome score between both groups also confirm this.

Due to the retrospective character of the study a detailed interview, why the patients did not want to undergo the revision reconstruction right after bone grafting was not possible. But Feucht et al. [13] highlighted the inferior expectations concerning return to sport, pain and the overall condition in patients with revision ACL reconstruction or previous operations. Therefore, the reduced functional approach and the improved functional status after the bone grafting could be another explanation for the rejection.

Besides subjective instability, some patients also presented with deficits in motion or locking of the knee. Before the bone tunnel grafting a routine diagnostic arthroscopy and resection and debridement of the previous graft and scar tissue was performed. During this procedure additional pathologies, like meniscal tears or severe cartilage defects, were treated. Therefore, some of the complained symptoms might be reduced only by the first surgery. Nearly all of the patients (8 out of 10) without revision ACL reconstruction had additional treatment during the first stage of operation. Although these patients did not complain about subjective instability or recurrent episodes of “giving way” - phenomena, clinical instability was clearly evident without revision ACLR.

In case of revision surgery, a thorough analysis of already utilized tendons, muscle strength and preferred sports is necessary. A variety of possible grafts for revision ACL reconstruction are described [14, 15]. According to the results of the MARS group [16], autografts are favorable compared to allografts with regards to re-rupture rate, clinical outcome scores and sports function. Contralateral hamstrings were also the most common tendons for revision ACL reconstruction in our study population.

In general, ACL revision surgery is challenging and postoperative reported functional outcomes are reduced compared to primary reconstruction [2, 17]. Nevertheless, a recent meta-analysis demonstrated a significant improvement for Lysholm score by revision ACL reconstruction from average 61.5 to 87.5 points. Results for Lysholm scores in the present study are slightly below these values, but according to Briggs et al. [18] a normal to nearly normal overall knee function is associated with a Lysholm score of 73 ± 15 points. From previous literature, the minimum detectable difference for Lysholm score is reported to be between 8.9 and 10.1 points [18, 19]. However, in the current study, the difference between the two groups was 4.3 point. Therefore, no significant or clinically relevant difference was observed between the two study groups. Also, no significant difference between both groups for Tegner score was evident. After bone grafting patients with and without revision ACL reconstruction reached a mean Tegner activity score of 4. In case of revision a mean Tegner of 5 is reported in recent literature [20]. However, Tegner activity scale includes level of sport activity and occupational function [18]. A closer look what is included in a level 4 of Tegner score is necessary for a further interpretation. Patients with a score of 4 could do moderate heavy work and could at least run two times a week. Interestingly, the Tegner score in persons with a “normal knee function” is located at mean of 5.7 [21].

The present study has several limitations. First of all, it is a retrospective case series with known limitations. Furthermore, the number of included patients, especially in the group of patients without revision ACL surgery, is limited. In fact, the calculated power of the current study based on the Lysholm score was 0.33. As most of the patients regularly undergo revision ACL surgery following bone grafting, it is almost impossible to obtain sufficient statistical power. However, the current study highlights, that not every patient requires or is willing to undergo further ACL reconstruction without compromising patient reported outcomes. Follow- up in present study is short term and maybe difference between both groups only occur in mid and long term follow-up. On the other hand, to the authors’ knowledge, this is one of the first studies to compare clinical outcome in patients with and without revision ACL reconstruction after bone grafting.

## Conclusion

Bone tunnel grafting due to revision ACL instability with or without second stage ACL revision surgery showed similar clinical results. Thus, in case of revision ACL instability precise patient selection for a renewed operative treatment is necessary.

## Abbrevations

ACL: Anterior cruciate ligament
CT: Computer tomography
IKDC: International Knee Documentation Committee Score
KOOS: Knee Injury and Osteoarthritis Outcome Score
LCL: Lateral collateral ligament
MCL: Medial collateral ligament
NS: Not significant
OBG: Only bone grafting
PCL: Posterior cruciate ligament
RACL: Revision anterior cruciate ligament
ROM: Range of motion
